# Simultaneous assessment and training of an upper-limb amputee using incremental machine-learning-based myocontrol: a single-case experimental design

**DOI:** 10.1186/s12984-023-01171-2

**Published:** 2023-04-07

**Authors:** Markus Nowak, Raoul M. Bongers, Corry K. van der Sluis, Alin Albu-Schäffer, Claudio Castellini

**Affiliations:** 1 Institute of Robotics and Mechatronics, German Aerospace Center (DLR), Münchner Str. 20, 82234 Weßling, Germany; 2 Department of Human Movement Sciences, University Medical Center Groningen, University of Groningen, Groningen, The Netherlands; 3 Department of Rehabilitation Medicine, University Medical Center Groningen, University of Groningen, Groningen, The Netherlands; 4 Department of Informatics, Technical University of Munich (TUM), Munich, Germany; 5 Assistive Intelligent Robotics Lab, Friedrich-Alexander-Universität Erlangen-Nürnberg (FAU), Erlangen, Germany

**Keywords:** Hand prosthesis, Machine-learning control, Myocontrol, Training, Single-case experimental design

## Abstract

**Background:**

Machine-learning-based myocontrol of prosthetic devices suffers from a high rate of abandonment due to dissatisfaction with the training procedure and with the reliability of day-to-day control. Incremental myocontrol is a promising approach as it allows on-demand updating of the system, thus enforcing continuous interaction with the user. Nevertheless, a long-term study assessing the efficacy of incremental myocontrol is still missing, partially due to the lack of an adequate tool to do so. In this work we close this gap and report about a person with upper-limb absence who learned to control a dexterous hand prosthesis using incremental myocontrol through a novel functional assessment protocol called SATMC (Simultaneous Assessment and Training of Myoelectric Control).

**Methods:**

The participant was fitted with a custom-made prosthetic setup with a controller based on *Ridge Regression with Random Fourier Features* (RR-RFF), a non-linear, incremental machine learning method, used to build and progressively update the myocontrol system. During a 13-month user study, the participant performed increasingly complex daily-living tasks, requiring fine bimanual coordination and manipulation with a multi-fingered hand prosthesis, in a realistic laboratory setup. The SATMC was used both to compose the tasks and continually assess the participant’s progress. Patient satisfaction was measured using Visual Analog Scales.

**Results:**

Over the course of the study, the participant progressively improved his performance both objectively, e.g., the time required to complete each task became shorter, and subjectively, meaning that his satisfaction improved. The SATMC actively supported the improvement of the participant by progressively increasing the difficulty of the tasks in a structured way. In combination with the incremental RR-RFF allowing for small adjustments when required, the participant was capable of reliably using four actions of the prosthetic hand to perform all required tasks at the end of the study.

**Conclusions:**

Incremental myocontrol enabled an upper-limb amputee to reliably control a dexterous hand prosthesis while providing a subjectively satisfactory experience. The SATMC can be an effective tool to this aim.

## Background

In our world tailored to interact using one’s hands people with upper limb absence use prosthetic limbs to overcome resulting challenges. Upper-limb prostheses have seen major technological advances in the last decade. Multi-articulated prosthetic hands with individual finger actuation are becoming more present and can be combined with (multi-articulated) actuated prosthetic wrists [1].

These technological advancements are accompanied by novel developments in myocontrol, which is the control of (prosthetic) devices using muscle signals, most commonly based on electromyography (EMG). These developments are manifold and include the distinction of up to 11 intended *actions*, such as *power grasp*, *pointing index* or *wrist flexion*, with a success rate above $94\%$ [2], the usage of high-density sensor matrices for control of up to 4 degrees of freedom (DOFs) of a robotic arm [3] or for decoding spike trains [4, 5], feature extraction based on deep learning [6] or the usage of different sensor modalities, such as forcemyography [7–10], ultrasound [11, 12] or electrical impedance tomography [13, 14]. Yet, the clinical standard since decades is a two-electrode control that uses a EMG-based switching command to cycle through the DOFs of the prosthetic setup [15]. Although many approaches, such as the ones mentioned in this paragraph, provide promising results, only few that are based on machine learning (ML) have reached the users [16, 17].

The process from initial algorithm development to applying the algorithm in daily living prosthesis use faces a number of challenges. An early involvement of the user is essential, since findings offline (without the user in the loop) do not translate to the online application (with the user in the loop) [18]. Hence, online testing with the user performing goal-reaching tasks has become the standard in evaluating the performance of a novel method [8, 19–22]. Moreover, the introduction of ML-based methods adds a further processing step to myocontrol. Instead of directly using sensor readings as control for prostheses, these signals are first processed and interpreted before they can be converted into control signals of the hand. This introduces a further layer of complexity for the user. Although measures have been taken to make this layer as intuitive and easy-to-use as possible [23, 24], studies have shown that ML-based methods require an extended training and learning phase [25, 26], which users can experience as exhaustive, potentially leading to abandonment [27–29]. A further aspect that is challenging for ML-based myocontrollers is the *limb-position effect* [30, 31]. It describes the issue that the measured EMG depends on the specific body posture, potentially leading to the myocontroller detecting another action then intended. Particularly ML-based myocontrollers suffer from this effect as already minor changes in the muscle configuration can have a significant influence on the recognition of an action [31].

*Incrementality* can be a solution to deal with these issues. Incremental ML methods allow the user to update or add new information to the training data, instead of retraining completely anew. This reduces calibration time significantly. Small and/or regular updates to an ML-based myocontroller in positions where it is required have been shown to improve performance of a myocontroller [25, 32–34].

However, existing validated assessment tools don’t explicitly take incrementality into account and are not tailored for use with ML-based myocontrollers. A recent overview has been provided by Kyberd [35].

We have taken inspiration from a number of validated assessment tools and developed the *Simultaneous Assessment and Training of Myoelectric Control* (SATMC) procedure. It can deal with incrementality and the specifics of novel myocontrol methods, allows the user to gradually improve and at the same time continuously assesses the myocontrol system. We have published a preliminary description and evaluation previously [36].

In this work we performed a long-term study involving one transradial amputee fitted with a multi-articulated prosthetic hand and a custom-built socket controlled by an incremental myocontroller based on *Ridge Regression with Random Fourier Features* (RR-RFF) [7, 8, 25, 32, 33]. Using the SATMC procedure allowed the participant to train how to use the incremental myocontroller, while simultaneously assessing the performance of the user in daily-living tasks. The goal of this study was to show that an incremental training protocol can be used to train a user to learn a complex myocontroller while at the same time assess the improvement of the user.

## Methods

### User study

After being thoroughly informed about the content and risks of the study, the participant (P) signed an informed consent form and agreed to participate. This study was formally approved by the host institution’s internal committee for data protection (ASDA 14/05 TOP 6.5 on 02.09.2014) and it followed the guidelines of the World Medical Association’s declaration of Helsinki. The male participant was 35 years old at the start of the study. He had undergone a traumatic transradial amputation of his left arm 11 years prior to the study. He routinely used a Variplus hand (Otto Bock GmbH) with a standard two-sensor control for opening and closing of the hand. He had neither experience with multi-articulated prosthetic hands nor with ML-based myocontrol. For the participation he received financial compensation for the cost of the commute and his time.

During the experiment two experimenters were present at all times. One person was the operator, who was concerned with supervising the myocontrol including updates, monitoring of the signals and assuring the correct completion of the protocol. A second person had a purely observational role making notes regarding the behaviour and manner of task execution.

The user study is based on *Single-Case Experimental Design* (SCED) [37–39], which provides guidelines for performing structured experiments involving only a small number of participants. Although less common in the field of prosthetics, a number of studies following these guidelines have been performed [40–43]. SCED-based studies can provide a high level of evidence, if carried out correctly [38].

### Prosthetic hardware

**Fig. 1:**
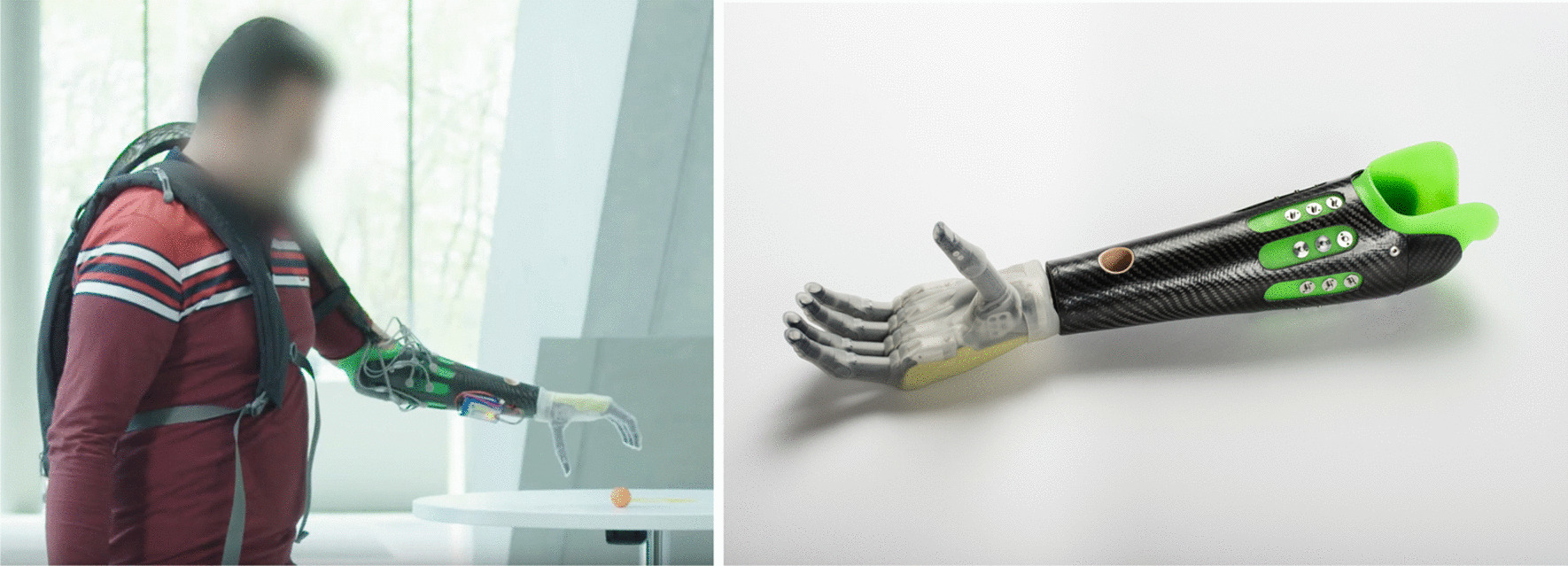
Prosthetic setup in our study; On the left: Participant P wearing custom-build hardware consisting of a small backpack housing hardware for data acquisition and wireless communication and a battery. On the right: Custom-build socket with eight snap-on electrodes uniformly distributed around the circumference of the stump. The prosthetic hand was the i-LIMB Revolution (Össur hf)

For the purpose of this study P was fitted with a custom-made prosthetic socket that could house eight myoelectric sensors. The design and the fitting were done by a certified prosthetist of Pohlig GmbH in Traunstein, Bavaria, Germany (part of Otto Bock GmbH). For the setup in this study eight 13E200=50 MyoBock sensors were used [44]. This is a larger number than the two-sensor arrangement of direct control, but eight sensors have already been successfully used in daily living as part of commercially available solutions [16, 17]. The electrodes were placed uniformly distributed around the circumference of the proximal forearm using snap-on domes. The most proximal snap-ons were placed 6*cm* from the medial epicondyle. The inter-dome distance spans 1.5*cm*. With this arrangement the electrodes cover the majority of the forearm muscles. These were embedded in the inner silicone layer of the design, while the outer layer was manufactured out of carbon fibre, see Fig. 1. Using custom-made electronics, the sensors were connected to the aforementioned snap-on domes. The communication between the sensors and the desktop computer used for computation was wired in the beginning of the experiment and wireless from session 24 onwards. The hardware was not optimised to fit in the confined space of the prosthetic socket. Hence, P was required to carry a small backpack with battery-powered electronics for reading the sensors and transmitting these readings to the desktop machine (Fig. 1, left-hand side). There was no additional weight on the prosthesis impacting the performance besides the socket and the prosthetic hand, which was an i-LIMB Revolution (Össur hf). The i-LIMB Revolution is capable of individual finger flexion for all five fingers and additionally thumb abduction.

### Incremental myocontrol algorithm

The basis of RR-RFF is *Ridge Regression*, which is linear regression with a regularisation parameter,

1 $$\begin{aligned} \varvec{\hat{y}} = \varvec{W}\varvec{x}{} \quad \text{with}{} \quad & \varvec{W} = (\varvec{X}^T \varvec{X} + \lambda \varvec{I})^{-1} \varvec{X}^T \varvec{Y}. \end{aligned}$$

The terms in Eq. (1) represent the predicted values $\varvec{\hat{y}}$, the regression weights $\varvec{W}$, the input $\varvec{x}$, as well as the regularisation hyperparameter $\lambda$ and the identity matrix $\varvec{I}$. $\varvec{X}$ and $\varvec{Y}$ are the collection of all data used for training and the associated target values, respectively.

RR-RFF is an extension of *Ridge Regression*, where the input $\varvec{x}$ is projected into a higher-dimensional space using a finite-dimensional approximation of a *Gaussian Kernel*,

2 $$\begin{aligned} \varvec{\phi } = \phi (\varvec{x})&= \sqrt{2}\cos (\varvec{\Omega } \varvec{x} + \varvec{\beta }), \end{aligned}$$

3 $$\begin{aligned} \varvec{\Phi } = \phi (\varvec{X})&= \sqrt{{2}/{D}}\cos (\varvec{X} \varvec{\Omega }^T + \varvec{\text{B}}), \end{aligned}$$

with

4 $$\begin{aligned} \varvec{\Omega }&\sim \mathcal {N}(0,\sigma ^2), \end{aligned}$$

5 $$\begin{aligned} \varvec{\beta }, \varvec{\text{B}}&\sim \mathcal {U}(-\pi ,\pi ), \end{aligned}$$

where $\sigma ^2$ is a hyperparameter and represents the variance of the Gaussian Kernel. This mapping $\phi : \mathbb {R}^d \rightarrow \mathbb {R}^D$ transforms the *d*-dimensional input space into a *D*-dimensional feature space. *D* is a further hyperparameter of the algorithm. Applying this transformation to Eq. (1) results in the final expression of RR-RFF,

6 $$\begin{aligned} \varvec{\hat{y}} = \varvec{W}\varvec{\phi }{} & {} \text{with}{} & {} \varvec{W} = (\varvec{\Phi }^T \varvec{\Phi } + \lambda \varvec{I})^{-1} \varvec{\Phi }^T \varvec{Y}. \end{aligned}$$

This non-linear mapping allows the algorithm to adequately fit data, where a linear mapping would not be sufficient. A detailed description of the underlying properties will not be covered here as previous publications already have done so [32, 45, 46].

An arbitrary number of electrodes can serve as input to the algorithm. The eight sensors used in this study are non-invasive and provide an already pre-filtered surface EMG (sEMG) signal, which is amplified, bandpass-filtered, and rectified onboard [44]. This signal was sampled at 100 Hz and further low-pass filtered with a 1^st^ order Butterworth filter with a cut-off frequency of 1 Hz. The resulting feature was the envelope of the sEMG signal and comprised the input to the RR-RFF-based myocontroller. The predicted output $\varvec{\hat{y}}$ of Eq. (6) was the individual DOFs of the prosthetic hand. These were the flexion of each of the five fingers plus the abduction of the thumb. By controlling each finger different actions can be composed, e.g. *power grasp*.

Furthermore, a number of features of RR-RFF were relevant in the context of this user study. First, based on previous studies, hyperparameters and modifications have been identified that allow for a fast and incremental update of the algorithm [32]. This feature is particularly relevant when dealing with the *limb position effect*. Due to incremental learning in positions, where the control becomes unstable, additional repetitions can be gathered, the algorithm can be updated and the execution can continue after a few seconds. Furthermore, the RR-RFF-based myocontroller predicts the individual DOFs of the prosthetic hand, e.g. *index flexion*, instead of action as a whole, e.g. *power grasp*. This feature allowed us to use incrementality for action training. That is, a new action that is added to an existing action set is represented by an additional configuration of the individual DOFs of the prosthetic limb and therefore does not require a further DOF in the vector of the target values $\hat{y}$. This feature allows the user to start with a minimal functional training and perform updates only when required and, therefore, reduces the initial calibration time of the myocontroller. An initial training can consist of only *rest* and *power grasp* and in a later update additional actions can be added, e.g. *precision grasp*.

Second, collection of training data is only done on the sustained part of action execution. This is the static part of the feature-data, when the user maintains an action with an approximately constant force. Often ramped training data is collected for regression-based algorithms [47–49]. This refers to the onset (and offset) of the feature-data, when transitioning from resting to an action. While there are benefits of using the dynamic part of the contraction [31], some users are not capable to follow a ramped signal closely [32, 50]. To avoid poorly labelled data, only the sustained part of an action is taken into account, which can be maintained for a few seconds. This has the added benefit that a screen displaying the visual stimulus is not required simplifying updates in positions and during tasks, where no screen is visible.

Third, the myocontroller is capable of *progressive forgetting*. Under the assumption that updates are required once a given situation has changed and/or the participant expresses different/improved sEMG signals, older training data becomes obsolete. The behaviour is that of a ring buffer, where the addition of an entry beyond the size of the buffer leads to the removal of the first/oldest entry. Since the training of the myocontrol is based on repetitions of actions, the size was set to five repetitions per action. This means that up to the fifth repetition the repetitions are added to the training data and therefore increasing the amount of training data. With the addition of the sixth repetition the chronologically first repetition will be removed. Therefore, after adding the fifth repetition to the training data the amount of data remains constant. This process applies to each action trained.

### Simultaneous assessment and training of myoelectric control (SATMC)

Based on the issues described in “Background” we formulated four aspects (A1–A4) that we deem important in the design of an assessment and training tool for ML-based myocontrol: *repeatability and increasing difficulty* (A1), *postural variation during tasks* (A2), *multiple actions per task* (A3), and *a short familiarisation time for the rater* (A4).

Among validated assessment tools none satisfies all four of these aspects. Exemplary from the most common tests for prosthetic control we evaluate the *Assessment of Capacity for Myoelectric Control* (ACMC) [51], the *Southampton Hand Assessment Procedure* (SHAP) [52], and the *Clothespin Relocation Test* (CRT) [53] considering the aspects A1–A4.

The ACMC is an observational assessment tool for prosthetic usage that can be performed in the home of a user or a room specifically designed to provide a household environment. Being able to observe a user in an environment as close as possible to daily living provides highly relevant insights in the validity of a given prosthetic system. As the tasks can be any activity of daily-living aspects A2 and A3 can easily be fulfilled. However, no specific guidelines provide structure to task repetition or increase of task difficulty (A1). Furthermore, professional training is required to draw proper conclusions purely from observations, making the ACMC less accessible (A4). Its current version is not tailored to multi-articulated prostheses [35].

The SHAP on the other hand is a test that can be administered with minimal training of the experimenter (A4) and only requires a suitcase of objects for its execution. The SHAP is a collection of tasks that are abstractions of activities of daily living (ADLs). They are performed in a seated position at a table and evaluated using an easy-to-use measure, the time to finish a task. The seated position only allows for limited assessment of issues arising from the limb position effect and therefore does not fulfil A2. The tasks have different levels of difficulty, however the limited options to vary tasks do not allow for a structured approach to increase difficulty (A1). Furthermore, the SHAP is comprised of unilateral tasks, which are all based on grasping actions (A3).

A good example of a test targeted at multi-articulated prostheses and complex tasks is the CRT. This test requires simultaneous activation of a prosthetic wrist and hand and thereby satisfies A3. As the name suggests clothespins need to be relocated from a horizontal bar to a vertical one, which in this case requires a rotation of the clothespins while maintaining a firm grip. Since the CRT consists of one task only, it offers rather little variability in its execution and difficulty (A1), but it contains postural variation (A2). Furthermore, only once a user is proficient in the use of their prosthesis the CRT can offer insight in the user’s capabilities. As no dedicated training of the experimenter is required A4 is satisfied.

The SATMC combines the advantages of the aforementioned assessment tools with added focus on aspects A1–A4. The following paragraphs describe how these aspects are implemented in the SATMC. They describe the *guidelines*, the implementation of structured *tasks* and a customised *experimental setup*.

#### Guidelines

An essential feature of the SATMC is a progressive increase in difficulty at a speed adaptable to the capabilities of the user (A1). This increase is two-fold. On the one hand each task has different levels of difficulty and on the other hand within the protocol we employ a stepwise increase in control complexity. The latter is realised by increasing the number of actions to control. In the beginning only two actions are available to the user. Starting with an action very commonly used, e.g. a hand close gesture/power grasp and a hand open / rest gesture. This initial action set already provides the functionality of common gripper prostheses.

The SATMC is organised in *sessions* and *phases*. A *session* is a collection of tasks administered as a closed unit. Per visit only one session is performed. A *phase* is characterised by multiple sessions with a specific action set and therefore spans multiple visits of the participant. Moving from one phase to another represents an increase in controller complexity as another action is added to the current action set.

**Fig. 2:**
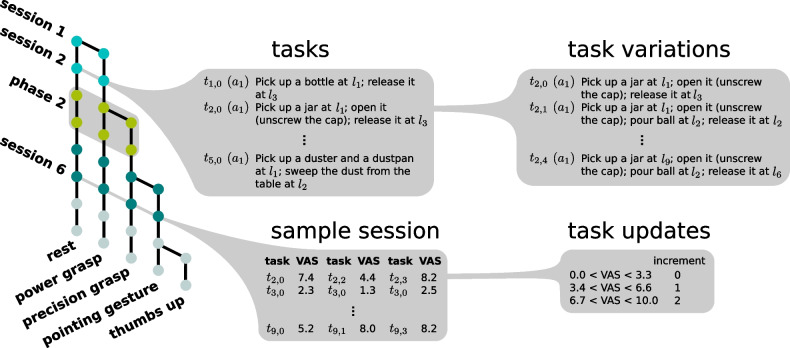
Diagram of the SATMC: Each bifurcation indicates the onset of a new phase, where an action is added to the existing ones and the tasks are updated. Sessions are visualised by rows spanning a set of actions of the same colour. Balloons *tasks* and *task variations* list exemplary tasks with exemplary task variations for a given session. Furthermore, balloon *sample session* gives a short overview of the process in an example session, where the tasks in the first column are executed, evaluated in column two and updated accordingly. The update is based on the VAS evaluation, see balloon *task updates*. This process is then repeated until 15 tasks have been performed and evaluated. $t_{i,j}$ represent variation *j* of task *i*. $a_{k}$ is action *k* and $l_{m}$ is landmark *m* in the study setup

In a session the user performs three repetitions of a set of five tasks. Each task has five variations of increasing difficulty, which are designed to fulfil aspects A2 and A3. Further details regarding tasks can be found in Paragraph “Tasks”. After a set of five tasks, the user is asked to self-evaluate their performance. For this purpose, we use a *visual analogue scale* (VAS), on which better or easier performance is rated higher. These self-evaluations determine the degree of difficulty for the next task variations. Based on an equal split of the scale, an evaluation of VAS 0–3.3 results in a repetition of the previous level of difficulty, while an evaluation of VAS 3.4–6.6 leads to an increase by one step and an evaluation of VAS 6.7–10 leads to an increase by two steps. These evaluations determine the next five variants of the tasks. Following their execution, this second set of task variants is evaluated determining the third and last five variants of the tasks. They are performed and evaluated, which then concludes one session with a total of 15 task executions. It is possible that in case of low VAS ratings a variant of a task is repeated three times within one session. Once a user becomes proficient in the performance with a given set of actions, a new phase of the study can be started. Two consecutive sessions, in which the self-assessment of all 15 tasks is in the range VAS 6.6–10 determines this point and a new action can be added to the existing set. Since the set of actions has been expanded the tasks have to be updated as well to ensure the usage of all available actions. An exemplary graphical representation of this process is given in Fig. 2.

Additionally to the self-assessment by the user, an easy-to-use measure has been chosen to assess the tasks in order to only require little to no training of the experimenter (A4). For this purpose, the *task completion time* (TCT) was selected. It has been shown that timing tasks is a key parameter for prosthetic use [54]. It is important to note that a focus was put on continuing a task rather than ending it prematurely due to erroneous behaviour. In case an object e.g. is dropped, rather than ending a task and counting this task as failed the user is encouraged to pick the object up again and continue with the task. This becomes particularly relevant in situations where the control algorithm reaches its limits, e.g. postures in which no training was performed. In these situations, where an execution is not possible due to poor performance of the myocontroller and retraining is required, the additional time spent on retraining is part of the task execution time and contributes to the overall evaluation. Therefore, algorithms that allow for a quick recalibration or even incremental learning will have shorter task durations in difficult situations.

Moreover, in order to reduce the burden on the user, they should be informed that the tasks are being timed, but they are not required to perform the tasks as fast as possible. The ADL-like tasks are not of a competitive nature. A fundamental principle in SATMC is repetition and improvement over time. The latter should still be evident in case the tasks are not executed as quickly as possible.

As it was mentioned in “User study” the SATMC follows SCED. Two central aspects are *direct replication* and the introduction of a *baseline*. The different phases of the SATMC correspond to *direct replication*, where each phase is a different condition the ML-based myocontroller is assessed in. To ensure a *baseline* throughout the administration of the SATMC one of the five tasks should be kept unchanged.

Following these guidelines, users can train their capabilities and the experimenter can assess the performance of user, prosthesis and myocontrol algorithm. The end of an experimental study can either be reached once this performance reaches its limits or by personal preference of the participant.

#### Tasks

Task design is influenced by aspects A1–A3 defined in “Simultaneous assessment and training of myoelectric control (SATMC)”. They mutual influence one another, as changes in posture (A2) and changes in the number of actions per task (A3) impact the difficulty of a task (A1). Height and rotational distance play an important role for grasp stability, i.e. at what height an object needs to be manipulated or over what height difference an object needs to be moved, and the extent of rotation required at the wrist. Furthermore, larger planar distances require for longer periods of stable grasping, which in turn make a task more difficult. These three distance measures (planar, vertical and angular) have been quantified as null, short, middle and long in order to compare different levels of task difficulty. Additionally, introducing subtasks in a given task is a further option to increase difficulty.

We have developed five variations for each task to reflect increasing levels of difficulty. These variations are indicated in the task number, e.g. $t_{2,3}$, which represents the third variation of task number 2. A list of tasks that we have developed according to the aforementioned considerations can be found in Table 1. The values of the three different distance measures per task variation can also be found in Table 1. This list of tasks is not exhaustive and not all tasks are required for the execution of the SATMC. Further tasks can be added keeping the aforementioned criteria in mind. In Table 1 tasks 10–12 have been omitted, since they were not used in the present user study. The tasks for each phase are selected by the person administering the SATMC. The set of five tasks should require all actions that have been trained so far, but not more, and should not be changed during a phase.

In the task descriptions in Table 1 several abbreviations are used. The landmarks $l_n$ can be found in the next paragraph describing the setup. The actions that are involved in each task are abbreviated by $a_n$ and correspond to

($$a_1$$)
power grasp,
($$a_2$$)
precision grasp,
($$a_3$$)
pointing gesture,
($$a_4$$)
preshaping for flat grasp (thumbs up), and
($$a_5$$)
flat grasp.

**Table 1:** Description of tasks

	Action(s)	Distance			
Task #	Involved	Planar	Vertical	Angular	Description
$t_{1,0}$	($a_1$)	Short	Null	Null	Pick up a bottle at $l_1$; release it at $l_3$.
$t_{1,1}$	($a_1$)	Middle	Short	Null	Pick up a bottle at $l_1$; release it at $l_4$.
$t_{1,2}$	($a_1$)	Long	Middle	Null	Pick up a bottle at $l_1$; release it at $l_9$.
$t_{1,3}$	($a_1$)	Short	Null	Middle	Pick up a bottle at $l_1$; pour water from it in a mug at $l_2$; release it at $l_1$.
$t_{1,4}$	($a_1$)	Middle	Middle	Middle	Pick up a bottle at $l_9$; pour water from it in a mug at $l_4$; release it at $l_9$.
$t_{2,0}$	($a_1$)	Short	Null	Null	Pick up a jar at $l_1$; open it (unscrew the cap); release it at $l_3$.
$t_{2,1}$	($a_1$)	Short	Null	Middle	Pick up a jar at $l_1$; open it; pour ball at $l_2$; release it at $l_2$.
$t_{2,2}$	($a_1$)	Middle	Middle	Middle	Pick up a jar at $l_1$; open it; pour ball at $l_2$; release it at $l_6$.
$t_{2,3}$	($a_1$)	Long	Middle	Middle	Pick up a jar at $l_8$; open it; pour ball at $l_2$; release it at $l_6$.
$t_{2,4}$	($a_1$)	Long	Long	Middle	Pick up a jar at $l_9$; open it; pour ball at $l_2$; release it at $l_6$.
$t_{3,0}$	($a_1$)	Short	Null	Null	Pick up a basket at $l_1$; release it at $l_3$.
$t_{3,1}$	($a_1$)	Middle	Null	Null	Pick up a basket at $l_1$; release it at $l_4$.
$t_{3,2}$	($a_1$)	Middle	Middle	Null	Pick up a basket at $l_5$; release it at $l_4$.
$t_{3,3}$	($a_1$)	Middle	Middle	Null	Pick up a basket at $l_5$; release it at $l_2$; take out object to $l_1$.
$t_{3,4}$	($a_1$)	Middle	Long	Null	Pick up a basket at $l_5$; release it at $l_2$; take out object to $l_9$
$t_{4,0}$	($a_1$)	Short	Null	Null	Pick up salami at $l_1$; bring it to chopping board at $l_2$; slice it with knife.
$t_{4,1}$	($a_1$)	Middle	Null	Null	Pick up salami at $l_3$; bring it to chopping board at $l_2$; slice it with knife.
$t_{4,2}$	($a_1$)	Middle	Null	Null	Pick up salami at $l_4$; bring it to chopping board at $l_2$; slice it with knife.
$t_{4,3}$	($a_1$)	Long	Middle	Null	Pick up salami at $l_9$; bring it to chopping board at $l_2$; slice it with knife.
$t_{4,4}$	($a_1$)	Long	Middle	Null	Pick up cutting board at $l_8$; bring it to $l_2$; pick up salami at $l_9$; bring it to chopping board at $l_2$; slice it with knife.
$t_{5,0}$	($a_1$)	Short	Null	Null	Pick up duster and dustpan at $l_1$; sweep the dust from the table at $l_2$.
$t_{5,1}$	($a_1$)	Middle	Null	Null	Pick up duster and dustpan at $l_1$; sweep the dust from the table at $l_4$.
$t_{5,2}$	($a_1$)	Long	Null	Null	Pick up duster and dustpan at $l_1$; sweep the dust from the table at $l_4$; chuck the dust out in a wastebasket at $l_6$.
$t_{5,3}$	($a_1$)	Long	Middle	Null	Pick up duster and dustpan at $l_7$; sweep the dust from the table at $l_4$; chuck the dust out in a wastebasket at $l_6$.
$t_{5,4}$	($a_1$)	Long	Middle	Null	Pick up duster and dustpan at $l_7$; sweep dust from the table at $l_4$; chuck dust in wastebasket at $l_6$; bring duster and dustpan back at $l_7$.
$t_{6,0}$	($a_2$)	Short	Null	Null	Pick up DLR cube at $l_1$; stack it on another DLR cube at $l_2$.
$t_{6,1}$	($a_2$)	Middle	Null	Null	Pick up DLR cube at $l_1$; stack it on another DLR cube at $l_4$.
$t_{6,2}$	($a_2$)	Long	Long	Null	Pick up DLR cube at $l_7$; another at $l_9$; stack it on another DLR cube at $l_2$.
$t_{6,3}$	($a_2$)	Middle	Null	Null	Pick up a checker at $l_1$; stack it on another checker at $l_4$.
$t_{6,4}$	($a_2$)	Long	Long	Null	Pick up a checker at $l_7$; another at $l_9$; stack it on another checker at $l_2$.
$t_{7,0}$	($a_2$)	Null	Null	Null	Fold towel at $l_2$.
$t_{7,1}$	($a_2$, $a_1$)	Short	Null	Null	Get towel at $l_3$; Fold towel at $l_2$.
$t_{7,2}$	($a_2$, $a_1$)	Middle	Middle	Null	Get towel at $l_3$; Fold towel at $l_2$; return to $l_9$.
$t_{7,3}$	($a_2$, $a_1$)	Middle	Long	Null	Get towel at $l_4$; Fold towel at $l_2$; return to $l_9$.
$t_{7,4}$	*NA*				
$t_{8,0}$	($a_2$)	Null	Null	Null	Pull the handle up to zip the jacket at $l_2$.
$t_{8,1}$	($a_2$)	Middle	Null	Null	Get jacket from $l_8$; place it at $l_1$; Pull the handle up to zip the jacket at $l_1$.
$t_{8,2}$	($a_2$)	Null	Null	Null	Wear a jacket with a zipper; pick up the zipper’s handle; pull the handle up to zip the jacket.
$t_{8,3}$	($a_2$)	Middle	Null	Null	Pick up jacket at $l_1$; Put jacket on; pick up the zipper’s handle; pull the handle up to zip the jacket.
$t_{8,4}$	($a_2$)	Middle	Null	Null	Unzip jacket at $l_1$; Pick up jacket at $l_1$; Put jacket on; pick up the zipper’s handle; pull the handle up to zip the jacket.
$t_{9,0}$	($a_3$)	Middle	Short	Null	Turn on the lights.
$t_{9,1}$	($a_3$, $a_1$)	Long	Middle	Short	Turn on the lights, grasp jar at $l_9$, put it back at $l_2$, turn the light off.
$t_{9,2}$	($a_3$)	Null	Null	Null	Dial a number at $l_1$ (vertical key).
$t_{9,3}$	($a_3$)	Short	Null	Middle	Dial a number at $l_1$ (horizontal key).
$t_{9,4}$	($a_3$, $a_1$)	Short	Null	Middle	Dial a number at $l_1$ (horizontal key); pick up handle; put it back down.
$\vdots$	$\vdots$	$\vdots$	$\vdots$	$\vdots$	$\vdots$
$t_{13,0}$	($a_3$)	Short	Null	Null	Roll small ball from $l_1$ to $l_2$.
$t_{13,1}$	($a_3$, $a_1$)	Middle	Null	Null	Roll small ball from $l_3$ to $l_2$, grasp it and put it at $l_8$.
$t_{13,2}$	($a_3$, $a_1$)	Short	Middle	Null	Roll small ball from $l_9$ towards you, let it fall, grasp it with the intact hand
$t_{13,3}$	($a_3$, $a_1$)	Middle	Middle	Null	Roll small ball from $l_5$ towards you, grasp it and put it in wastebasket at $l_6$
$t_{13,4}$	$NA$				

**Fig. 3:**
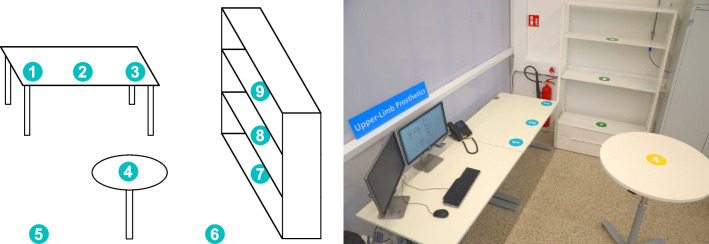
Overview of the setup used in the SATMC; a sketch on the left and the implementation in our laboratory on the right. Numbers in the setup indicate landmarks $l_n$, which are used in task descriptions

$a_n$ correspond to actions: ($a_1$) power grasp, ($a_2$) precision grasp, ($a_3$) pointing gesture, ($a_4$) preshaping for flat grasp (thumbs up), and ($a_5$) flat grasp; $l_n$ corresponds to landmarks described in Sect. “Setup” and can be seen in Fig. 3. Distance cut-offs are based on the setup and DOF usage. planar: short—only on rectangular table, middle—between rectangular table and round table or between shelf and round table, long—beyond that; vertical: short—between rectangular table and round table, middle – involving one level on the shelf, long—involving two levels on the shelf; angular: short— involving some rotation at the wrist level, middle – involving up to 90$^{\circ }$ rotation at the wrist level (supination or pronation), long—involving up to 90$^{\circ }$ rotation at the wrist level (supination and pronation). Note that tasks 10–12 are not presented since they were not used in this study

#### Setup

An overview of an instance of a setup with landmarks $l_n$ indicated as numbers *n* can be found in Fig. 3. These landmarks can be described as follows:

($$l_1$$)
on the rectangular table, straight in front of the participant
($$l_2$$)
on the rectangular table, half a meter laterally towards the intact hand.
($$l_3$$)
on the corner of the rectangular table.
($$l_4$$)
on the round table.
($$l_5$$)
on the ground, one side of the round table.
($$l_6$$)
on the ground, other side of the round table (wastebasket).
($$l_7$$)
on the shelf, lower level ($\sim$ 0.20 m above the ground).
($$l_8$$)
on the shelf, middle level ($\sim$ 1.00 m above the ground).
($$l_9$$)
on the shelf, top level ($\sim$ 1.80 m above the ground).

The participant is seated in front of $l_1$ at the beginning of a task. Based on these landmarks we approximated the difficulty in terms of planar and vertical distance, see Table 1. For example, picking up an object at $l_1$ and releasing it at $l_2$ is easier than picking it up at $l_1$ and bringing it to $l_9$.

**Fig. 4:**
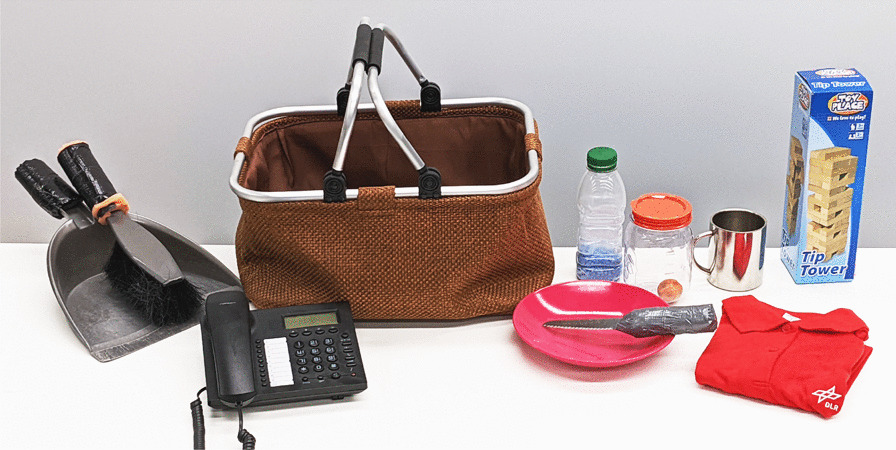
Objects used in the user study; from left to right: duster and dust pan, phone, basket, bowl with knife, bottle with “fluid”, jar with ball, shirt to fold, mug, and Jenga tower

Each task requires some objects to manipulate or move around. A set of objects required for the tasks in Table 1 can be found in Fig. 4.

### Analysis

Additionally to the primary measures TCT and VAS, we recorded sEMG-data for the entire duration of the experiment and logged each algorithm update with sEMG-data and timing for further evaluation.

Training data can be evaluated using common measures of data properties, e.g. the Separability Index (SI) and the Repeatability Index (RI) [21, 55, 56]. SI is a measure of cluster separation, where the distance between cluster centroids is weighted with the spread of the clusters.

7 $$\begin{aligned} \text {SI} = \frac{1}{n}\sum _{i=1}^{n} \left( \frac{1}{2} \sqrt{\left( \mu _i-\mu _{ci}\right) ^T S^{-1} \left( \mu _i-\mu _{ci}\right) } \right) , \end{aligned}$$

with *n* representing the number of actions, $\mu _i$ the centroid of action *i*, $\mu _{ci}$ the centroid of the most conflicting action for action *i* and $S=\frac{S_i + S_{ci}}{2}$ with $S_i$ and $S_{ci}$ representing the covariance of the aforementioned two corresponding actions.

RI usually compares the feature-data collected during training with the feature-data from the testing phase. Since the execution of the tasks in the SATMC is rather free, there is no ground truth in the testing phase that can be used for this comparison. As an alternative, we compare the repetitions of an action that are used to train the myocontrol, which in turn provides information on the data consistency between repetitions. The RI is a measure of difference between these repetitions per action, i.e. a distance measure of the repetition centroid weighted with the spread of the repetitions.

8 $$\begin{aligned} \text {RI} = \frac{1}{n}\sum _{i=1}^{n} \frac{1}{\left( {\begin{array}{c}r_i\\ 2\end{array}}\right) } \sum _{\begin{array}{c} j=1\\ k=1\\ j \ne k \end{array}}^{r_i} \left( \frac{1}{2} \sqrt{\left( \mu _{i,j}-\mu _{i,k}\right) ^T S^{-1} \left( \mu _{i,j}-\mu _{i,k}\right) } \right) , \end{aligned}$$

with *n* representing the number of actions, $r_i$ the number of repetitions for action *i*, $\mu _{i,j/k}$ the centroid of repetitions *j*/*k* of action *i*, and $S=\frac{S_{i,j} + S_{i,k}}{2}$ with $S_{i,j}$ and $S_{i,k}$ representing the covariance of two different repetitions of action *i*. The measures SI and RI were calculated only using training data.

Throughout the study sEMG-data was gathered during task execution together with the parameters and hyperparameters of the RR-RFF-based algorithm. Since the myocontroller is based on *Ridge Regression*, the least-squares formulation in Eq. (1) can be interpreted from a Bayesian perspective [57, 58]. Based on a new sample of data $\varvec{x}_{n+1}$, we can not only predict a single value $\varvec{\hat{y}}_{n+1}$, but also get information about the uncertainty of the predicted value. For this purpose, the *predictive distribution* is required

9 $$\begin{aligned} f(\varvec{\hat{y}}_{n+1}|\varvec{x}_{n+1},data), \end{aligned}$$

with *data* representing all samples ${x}_i$ and labels ${y}_i$ used for the calculation of the ML model. For *Ridge Regression* a closed form for the predictive distribution can be found. It follows a normal distribution with mean $\mu _{n+1}$ and variance $\sigma _{n+1}^2$

10 $$\begin{aligned} f(\varvec{\hat{y}}_{n+1}|\varvec{x}_{n+1},data) \sim \mathcal {N}(\mu _{n+1}, \sigma _{n+1}^2), \end{aligned}$$

with

11 $$\begin{aligned} \mu _{n+1}&= \left(\varvec{X}^T \varvec{X} + \frac{b}{a} \varvec{I}\right)^{-1} \varvec{X}^T \varvec{Y} \varvec{x}_{n+1}, \end{aligned}$$

12 $$\begin{aligned} \sigma _{n+1}^2&= \frac{1}{a} + \varvec{x}_{n+1}^T (a \varvec{X}^T \varvec{X} + b \varvec{I})^{-1} \varvec{x}_{n+1}. \end{aligned}$$

For a full derivation of $\mu _{n+1}$ and $\sigma _{n+1}^2$ we refer the interested reader to Bolstad and Curran [57].

From Eq. (11) we can see that the mean is equal to the predicted value from Eq. (1). The variance $\sigma _{n+1}^2$ allows us to evaluate the uncertainty of a predicted value, where high values represent high uncertainty and low values low uncertainty. Therefore, we can assess given the *data*, whether an action has been predicted with a high or a low confidence.

## Results

Our participant was followed for 13 months, during which P performed 31 sessions. The sessions took place once per week or every two weeks and lasted between 30 min and 2 h. A longer gap of three months occurred between sessions 22 and 23. Over these 31 sessions we attempted four different phases (characterised by an increase in number of actions), of which one was unsuccessful (*precision grasp*). As a baseline a task was needed that was not too complex but useful and it needed to fit the possibilities provided after initial action training. To this end, we selected task $t_{2,0}$ from Table 1 as a baseline measure. Furthermore, in order to compare the incremental ML-based myocontrol to the standard two-sensor myocontrol a second baseline measure was introduced. This was a single session (session 20) where P used his own prosthesis, which is controlled in this manner.

As a further note, at the beginning of each new session the ML model from the previous session was reloaded. The training data was only updated, when it was required and either asked for by the participant or initiated by the experimenters. This is based on our idea of incrementality, where only minimal initial ML training is performed and changes or uncertainties are dealt with by deliberate updates.

### Protocol overview

**Fig. 5:**
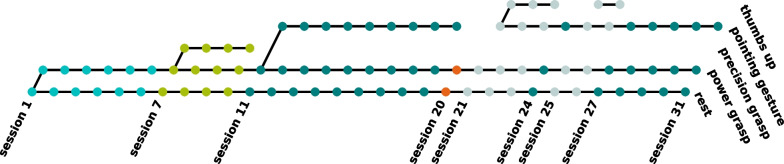
Overview of all sessions and phases present in the user study. Colours indicate different phases. In session 20, highlighted in orange, P performed tasks from the SATMC with his own prosthesis using direct two-electrode control

Figure 5 shows the process of the SATMC indicating each session and all actions that were attempted during the different phases. In session 7 the *precision grasp* was introduced, while from session 11 onwards said action was no longer part of the action set. P encountered difficulties with distinguishing the *power grasp* and *precision grasp* reliably. This became evident to the experimenters in terms of heavy jitter and instability in the myocontrol. Therefore, it was decided to consider this phase (phase 2) failed and P switched to a different grasp that was considered to be more likely to create a distinguishable action set, i.e. *pointing index* (phase 2’).

After reaching the end of phase 2’ P performed the second baseline in session 20 by using his own prosthesis, a myoelectric gripper. The tasks performed therein, were the ones from phase 2’. This session is highlighted in orange.

**Fig. 6:**
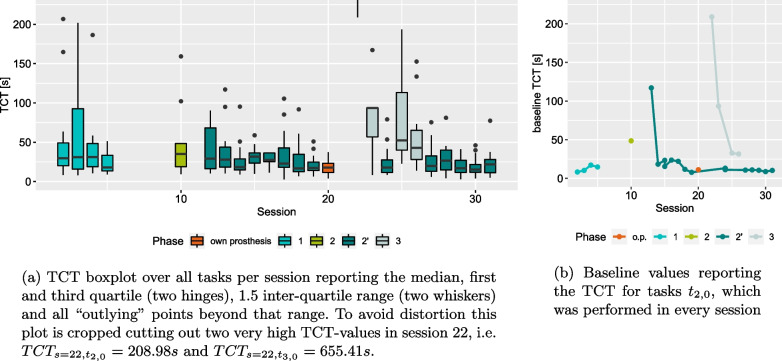
Primary assessment measure TCT with boxplot over all tasks per session and baseline values for task $t_{2,0}$

**Fig. 7:**
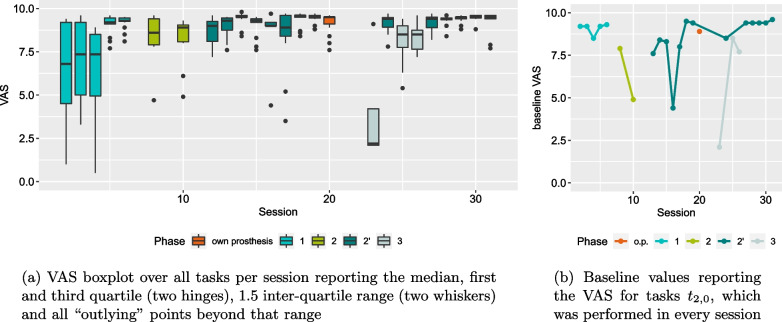
Self-assessment measure VAS with boxplot per session and baseline values for task $t_{2,0}$

### Timing evaluation

TCT was measured from the beginning of a task to its end, including potential missteps and / or updates to the controller. A summary of the TCT across the full user study can be found in Fig. 6. We can see an improvement within phases. Particularly, phases 2’ and 3 show a reduction in baseline TCT from session to session until reaching a plateau, see Fig. 6b. For phase 1 the trend for baseline TCT is slightly positive. Taking the plateau area of phase 2’ into account these values seem to be on a similar level. As phase 2 has only one measurement, no trends can be seen. However, the single value is higher than in phases 1 and 2’, which could indicate issues in task performance.

The TCT boxplots in Fig. 6a are in line with what is shown in Fig. 6b. Earlier sessions of phase 1 and 3 seem to have higher TCT values and a higher variance, which then drops towards the end of the phases. The trend seems to be less prominent in phase 2’, yet earlier sessions seem to have slightly higher values. Phase 2 again has only one measure, which is on a similar yet slightly higher level than the initial values of phase 2’ taking into account the outlines of the boxplot.

Additionally, we can see from the plots in Fig. 6 that the TCT with the incremental myocontroller is on a comparable level with P using his own prosthesis. The comparison should be drawn to phase 2’, since it involves the same tasks.

### VAS self-assessment

The self-assessment using a VAS followed a similar behaviour as TCT, see Fig. 7. Here the satisfaction was lower in the earlier parts of a phase than towards the end. This is particularly evident in phase 1 and 3. Phase 2’ contained a session that was particularly unsatisfying to P in the baseline task, see session 16 in Fig. 7b. Considering Fig. 7a, it seems that only this particular task was unsatisfying, since the remaining ones were evaluated similarly to the previous and following sessions and the VAS value for the baseline task was considered an outlier in the boxplot.

In general, the VAS assessment tended to be rather positive with $89\%$ of its values above 7.5 and a median of 9.3. Notable exceptions were the very early sessions of phase 1. The VAS values varied heavily within one session. Starting with session 5 the self-assessment became more consistent with higher values.

All individual VAS self-assessments can be found in Table 2.

### Updates

**Fig. 8:**
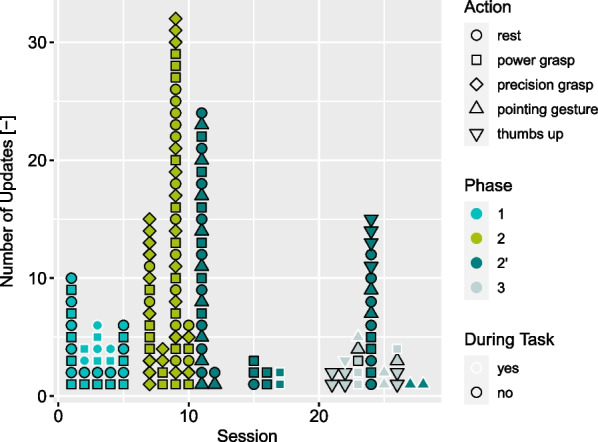
Updates of the myocontrol per session. Each symbol represents one update of an action. The shape indicates the respective action, the colour during which phase the update was performed and white or black outlines indicate, whether the update was performed during a task or in between tasks, respectively

The number of updates per session can be found in Fig. 8. It depicts how many of those updates were required during the performance of a task and what action was updated.

In phase 1 regular updates were required, which indicates a level of uncertainty in a situation where only *resting pose* and *power grasp* were required. The introduction of a further action in phase 2 increased the number of updates required even further. This shows the difficulty in finding a stable control for the action set of *resting post*, *power grasp* and *precision grasp*. Eventually, this phase was aborted and after the changes to the action set, a functional training data set could be found within one session. After 24 updates in session 11 only very few additional updates were required throughout the rest of phase 2’. Notable exception here is session 24 where a retraining with 15 updates occurred. Due to an error of the experimenter a full retraining was initiated, which would not have been required. Phase 3, where the *pre-lateral grasp* was introduced, provides a further indication of confidence in the navigation of the novel myocontroller. Only five repetitions of the newly added action were required to successfully perform tasks. Compared to phases 1 and 2 the number of additional updates was rather low.

Furthermore, we would like to point out that due to the myocontrollers capability to forget obsolete training data, the amount of training data used per action was almost constant throughout the user study. The limit for repetitions per action was set to 5. For phases 1, 2 and 2′ this level was already reached in the first session of a new phase, while for phase 3 this level was reached in the second session of the phase.

### EMG-data measures

**Fig. 9:**
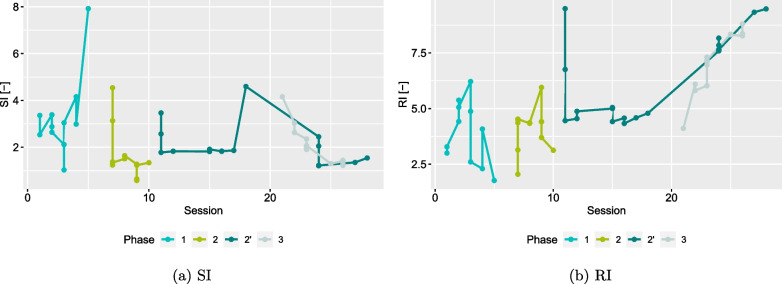
SI and RI after every update to the myocontroller. Coloured lines indicate the changes within one phase. Multiple updates per session were possible resulting in multiple points per session. Not every session required updates, which led to gaps in the visualisation

Figure 9 shows the evolution of SI and RI over the course of the entire study. Different phases have been colour-coded and the individual values of a phase were connected with a line to better visualise the changes within each phase. For several sessions more than one value is reported. Every time a model update had been performed, SI and RI were re-evaluated. In case there were several updates per session, all values are reported.

As we have mentioned in “Incremental myocontrol algorithm”, for our controller we implemented *progressive forgetting*. Obsolete repetitions of an action were discarded and therefore both SI and RI should converge to an optimal value for the user without the influence of obsolete data. As both SI and RI are measures of distance, SI should *increase*, signifying better separability between actions and RI should *decrease*, signifying higher repeatability and consistency in controlling one’s muscles.

In phases 1 and 2 both SI and RI did not seem to follow a clear trend. Phase 1 ended with positive developments from a theoretical point of view, i.e. a large increase in SI and considerable drop in RI. Phase 2 started with a high separability, dropped significantly and then remained at rather low values. RI started low, increased and expressed a varying behaviour that did not resemble a clear trend. The initial decrease of SI and increase of RI were theoretically negative developments. Phase 2’ started with both high SI and RI and then dropped within the first session. In the development of the RI a trend towards higher values became evident. For the SI we can see a plateau area followed by a large jump, after which a trend to lower values can be seen. This trend continued throughout phase 3 for both SI and RI.

**Fig. 10:**
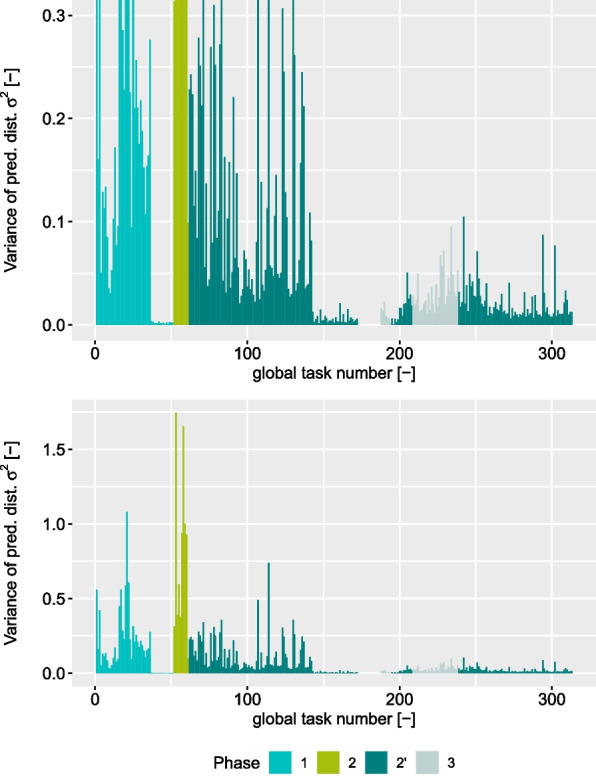
Variance of the predictive distribution $\sigma^2_{\text{pred}}$ averaged per task. Colour-coding indicates the phase of the experiment. The top plot has been cropped to better visualise the low end of the scale

Furthermore, we have calculated the predictive distribution for each data sample in each task of our study. Figure 10 shows the mean variance of the predictive distribution $\sigma^2_\text{pred}$ for each task in chronological order. Different phases of the experiment have been highlighted with different colours.

Phase 1 started with higher variance until task 37 in session 5, where a large drop can be seen. Thereafter the remaining tasks of phase 1 were performed with very low $\sigma ^2_\text{pred}$ indicating high consistency in the expressed control signals by P. After the transition to phase 2 the highest $\sigma ^2_\text{pred}$-values in the entire study can be seen. Neither a drop nor a considerable decrease was evident within this phase. The values represent a high level of uncertainty in P’s control and eventually this phase was considered failed. The change in the action set that came with phase 2’ led to decreased, yet still rather high values of $\sigma ^2_\text{pred}$. These remained consistent until task 143 in session 18, where a second considerable drop can be noticed. After the second drop there were no higher values for the rest of the study. This is even true after introducing a further action in phase 3.

**Table 2:**
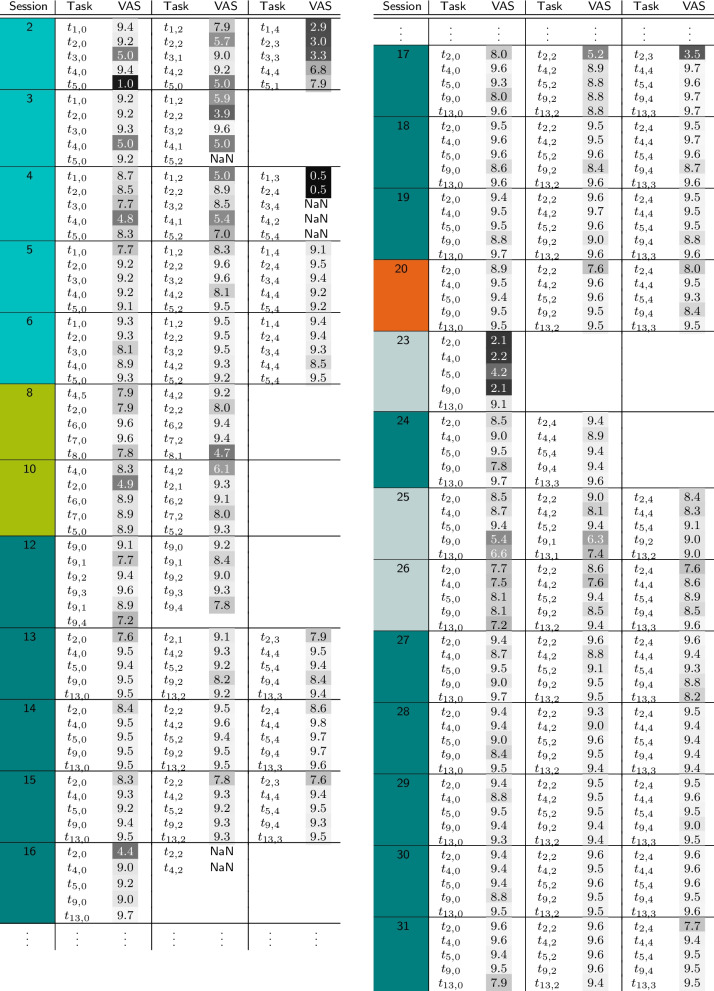
Self-assessment of all tasks using a VAS

VAS values are colour-coded between black $\text {VAS} = 0$ (poor evaluation) and white for $\text {VAS} = 10$ (good evaluation); the colour in the *session* column indicates the phase

## Discussion

Using an incremental myocontroller and the SATMC P was able to learn to reliably control four actions performed by a multi-articulated hand prosthesis in daily-living tasks. At the same time, the SATMC showed its capabilities to monitor P’s progress and to assess the performance of user and myocontroller. We were able to observe improvement within phases, improvement over the full study, identify failed phases, show the benefit of incremental myocontrol, and show comparable performance to using standard two-sensor control.

For both primary measures, TCT and VAS, we can see a positive development within phases. These trends can be seen particularly in the baseline task. The improvement within phases can also be seen in the predictive variance $\sigma ^2_\text{pred}$. Here, phases 1 and 2’ are of particular interest as in both cases a substantial drop can be seen. These measures indicate that the beginning of a phase required more effort and learning from P and within a few sessions improvement could be observed. Interestingly, the improvement in session 5 of phase 1 can be seen in both VAS and $\sigma ^2_\text{pred}$. The VAS self-assessment until session 4 showed considerable variance indicating a varying level of satisfaction with the performance. High $\sigma ^2_\text{pred}$-values indicate uncertainty in the myocontrol, as well. With the update in session 5 $\sigma ^2_\text{pred}$ and the variance of the VAS self-assessment both decreased. The average VAS for session 5 was very high, which indicated satisfaction and low $\sigma ^2_\text{pred}$ indicated high certainty in the usage of the myocontrol. This suggests that with the update in session 5 a suitable training dataset had been found and the lack of further updates indicated a stable and reliable myocontrol. This initial period of the study could have been an explorative period for P. Since P is a user of a prosthesis with direct control, switching to ML-based myocontrol could have initially required a high effort. Note that from the outset the myocontroller of the experiment was different from the myocontroller P used in his daily life.

The following phase 2 showed that an increase in myocontroller complexity required further training. However, the choice of action proved to be too demanding and a switch in the action set was required to continue with the user study.

Phase 2’ started similarly to phase 1. Decrease in TCT and increase in VAS values in the first sessions indicated improvement in the beginning, although with lower variance in the self-assessment as in phase 1. A further milestone marks session 18: after task 143 there was a substantial second drop in the variance of the predictive distribution $\sigma ^2_\text{pred}$. Both instances where $\sigma ^2_\text{pred}$ dropped considerably and remained low for a certain period exhibited a considerable increase in SI, as well. The RI on the other hand dropped with the first $\sigma ^2_\text{pred}$-drop and remained on a similar level with the second one. For SI, these are the two largest changes in the entire user study and seem to align very well with good performance. However, the remaining trend of SI towards lower values following the increase does not support the claim of correlation between good performance and a high SI [55, 59, 60]. The very last model used in the study even had a lower SI than the value before the drop in $\sigma ^2_\text{pred}$. Although non-conclusive, these findings are in line with what has been reported in literature regarding SI and RI and other offline measures [18, 20, 21].

Since after the second $\sigma ^2_\text{pred}$-drop, there were no tasks with high values for $\sigma ^2_\text{pred}$ for the rest of the study, this could indicate the beginning of another period for P. It could be argued that at this point P became proficient in the usage of ML-based myocontrol and an understanding of the myocontroller was established. The addition of a further action in phase 3 did not lead to uncertainty in the usage of the myocontroller. Yet P needed to adapt to the new myocontroller, which is apparent from the improvements in baseline TCT and baseline VAS, see Figs. 6b and 7b.

These two jumps could indicate three different periods in the improvement over the course of the study. First a familiarisation period, followed by a learning period and ending with a proficient adaptation period.

Two further points support the notion of reaching a proficient state. First, the performance in the second baseline measure, session 20 with P’s own prosthesis, is on a comparable level as the ML-based myocontroller. Under the assumption that P is proficient with his own prosthesis he could have reached a certain level of proficiency with the ML-based myocontroller as well. Switching from a familiar control modality to a more complex, yet more capable one can initially result in a reduced performance [40]. Even after 7 training sessions it was reported that people achieved better results with their own prothesis than with ML-based ones [26]. Second, the erroneously performed retraining in session 24 did not appear to have an impact on the performance, i.e. TCT, VAS or $\sigma ^2_\text{pred}$. One could argue that P’s performance didn’t originate from *accidentally* good data, but that P learned to *consistently* produce good signals to pilot the prosthesis and myocontroller.

### Incrementality

Incrementality played a key role in learning to use the myocontroller and in dealing with challenging situations.

There was no need for a separate training of sEMG-signals focused on separateness and repeatability, a process that commonly is required in learning to use a ML-based prosthesis. Exemplary duration of this process is 7–10 h over 5–7 session [26, 28]. The exact values vary considerably based on the individual person. Taken the two milestones of P in session 5 (after 1 month) and 18 (around the 7th month) into account, the training time can be considered longer. However, prosthesis fitting and training is most important in the first six months after amputation [61]. The fact that from the first session training involved functional tasks while wearing a prosthesis, could support prosthetist acceptance.

During P’s training, an update consisted of 2.7 repetitions on average, which includes the instances of full retraining. A full retraining would consist of five repetitions per action. In phase 1 this would be 10 repetitions and 20 repetitions in phase 3. This results in a considerable amount of time saved on individual updates when using an incremental myocontrol algorithm. Furthermore, updates were often asked for by P to make a certain action more stable or improve the performance in a specific situation. In our opinion, the threshold for issuing a small update is lower than issuing a full retraining. This could lead to faster learning and a better adaptation, and thus faster improvement in performance.

The combination of training only on the sustained part of an action and incremental updates further helped in dealing with the limb position effect. Instead of initially training in multiple positions to cover all required postural variations, updates could be issued in challenging positions only when required. Training on the sustained part of an action does not require the participant to follow a specific trajectory, however, an action has to be maintained at a strong but comfortable level of force. This allowed P to maintain exactly in the pose, where the myocontroller reached its limits, and update the training data with highly specific information to improve the myocontroller.

The capability of the RR-RFF-based myocontroller to add actions incrementally, reduced the calibration effort for P additionally. This effect became relevant at a later point in the user study, when P transitioned from phase 2’ to phase 3. Instead of requiring a full retraining only the new action had to be updated and P could continue with performing tasks.

A further testament to the robustness of the RR-RFF-based myocontroller is the fact that over the course of the study there were several instances where P did not require any update over several sessions. Hence, for multiple visits involving donning and doffing of the prosthesis no changes to the myocontroller were required and all tasks could be performed satisfactorily. In addition to that we want to highlight that no initial training after donning the prosthesis was issued at the beginning of a session.

### SATMC protocol

ML-based myocontrollers are intuitive in terms of the type of sEMG-signals that are required for training the algorithm. However, learning to pilot such a myocontroller reliably has proven to be challenging, lengthy, and not necessarily intuitive for many users. The SATMC appears to be a promising tool for assessing ML-based myocontrollers and training users in their usage. Supported by the structured multi-phased approach a gradual improvement was possible for P. Due to the usage of tasks of different levels of complexity, training was possible at a level comfortable for the participant. In “Simultaneous assessment and training of myoelectric control (SATMC)” we have formulated four aspects that should be fulfilled for an adequate assessment and training of ML-based myocontrol. These were repeatability and increasing difficulty (A1), postural variation during tasks (A2), multiple actions per task (A3), and a short familiarisation time for the rater (A4). In the user study P attempted scenarios with different levels of difficulty. Expressing a level of satisfaction through the VAS assessment confirms that A1 was successfully implemented. A number of tasks involved larger distances that needed to be covered, i.e. wrist rotation and height differences. These variations adequately cover the postural variation required in A2. Changes between different grasps were part of several of the tasks, which satisfied A3. Regarding A4, easy to acquire measures, i.e. VAS and TCT, have been introduced, which simplified the tasks of the experimenter. However, a number of errors occurred on the side of the experimenter, where instructions were not given correctly. As the SATMC has grown to cover as many relevant features as possible, the complexity of performing a study using the protocol increased over its development. Here, we believe that the initial level of simplicity intended for the SATMC has not been fully reached.

During the user study and in its evaluations two potential improvements to the SATMC have been identified. First, as stated in “User study” no initial training in a session was required, since the model from the previous session could be reloaded and reused directly. This beneficial feature is not reflected in any measure besides the number of updates. A solution could be adding to the 15 tasks an initial *calibration stage* that is timed and in case no initial training is required set to 0*s*.

Second, since phase 2 was considered failed, a measure to determine at what point a phase can be considered failed could be useful. One option could be based on the VAS evaluation of the user. However, the evaluation of sessions 8 and 10 in phase 2 was overly positive^1^. A second option could be a threshold on the variance of the predictive distribution $\sigma ^2_\text{pred}$. The highest values of $\sigma ^2_\text{pred}$ were measured in phase 2. This is only feasible, if the ML method allows for the calculation of the predictive distribution. Another option could be to use the number of updates required during a session. The failed phase contained the session with the highest number of updates in the entire study. A threshold on the number of updates could help identify failed phases.

With the structure and repetition that the SATMC introduces, we also introduce the risk of learning specific tasks rather than acquiring general motor skills. At the beginning of a phase five tasks are chosen by the person administering the SATMC and in each session the participant starts with the basic variants of these tasks. However, the fact that at the beginning of phase 2’ P only required a few repetitions to learn a new action compared to many repetitions in all previous phases, could indicate motor skill acquisition rather than task-specific learning, see Fig. 8. On the other hand, we also introduce variability with the SATMC. Depending on the skill of the participant different variants of these tasks are executed throughout a phase. Earlier session will likely involve easier task variants, while the last session will involve the most difficult ones. This variance potentially affects our primary measure TCT. Considering Fig. 6, we can see initially large values of TCT for phases 2’ and 3 followed by a plateau area. The low variance in the plateau area could indicate that influence of the task variants is small.

With these improvements we see a high potential of the SATMC to be applied in clinical use. Since training and assessment are both part of the SATMC training the participant to produce good sEMG-signals and functional assessment of prosthesis both occur at the same time. The user would start earlier with performing tasks with their prosthesis, which could have beneficial effects on motivation and acceptance. Furthermore, a number of steps in the SATMC can be automated as they follow strict guidelines, see “Guidelines”. This would reduce the burden on the person administering the SATMC and therefore increase the clinical applicability. In addition, the SATMC is not restricted to training the use of hand prostheses but can also be used to train more proximal prosthesis joints.

### Limitations

During the analysis of the results we were able to identify some limitations of the user study with P. In phases 2’ and 3 the SI exhibited a trend towards values indicating poorer training data quality, yet the participant improved and was more satisfied. As SI and RI are pure *offline* measures and TCT and VAS are *online* measures (with the user in the loop), a mismatch between them is a common phenomenon. A further possible explanation could be the way training data was gathered. The user is encouraged to update the myocontroller, when instabilities occur in the control. These instabilities could originate from changes in the muscle and limb orientation, i.e. limb position effect. An update will therefore contain data that is rather different from what was present in the training data before the update. This could lead to an increase in RI, since the update is labelled with the action that was updated without taking specific information of the position into account. A larger spread of the action cluster (containing all repetitions) would be the result and hence potentially lead to a lower SI. In general, a higher level of repeatability, as in being able to precisely reproduce a muscle signal, is a desirable feature. However, based on the training protocol RI and SI could potentially reflect a different measure than the intended separability and repeatability. On the other hand, both drops in the values of the variance of the predictive distribution $\sigma ^2_\text{pred}$ coincided with an increase in SI as one would expect.

Furthermore, we have identified some general improvements for the SATMC. A comparison to different validated assessment method, such as the ones described in In “Simultaneous assessment and training of myoelectric control (SATMC)”, would have strengthened the results of this user study. The administration of validated assessment tools at regular intervals of the user study, e.g. at the beginning and end of a phase, would have provided further insights in the validity of the SATMC. We see such an addition as useful for future studies based on the SATMC.

Additionally, some unfortunate mistakes by the experimenter were made during the user study. For one, phase 2’ was continued longer than it should have been. In sessions 13 and 15 the VAS scores were evaluated wrongly, which led to single-step increases in task variants instead of two-step increases. A correct decision in either of these sessions would have led to an earlier successful conclusion of phase 2’. Additionally, experimenter errors occurred in session 24, session 27 and the session thereafter: the experimenter gave instructions regarding the wrong phase. While the instructions were instances of phase 2’, they should have been instances of phase 3 according to the SATMC guidelines. Although unfortunate, in our opinion additional repetitions of a phase should not severely impact the overall conclusions drawn from this study. We believe that all errors regarding wrong VAS evaluation or wrong phase selection could be avoided by automating the SATMC. This could be achieved in form of a specific software that provides directions based the data acquired. This would also be a step towards aspect A4, defined in the beginning of “Simultaneous assessment and training of myoelectric control (SATMC)”. On the other hand, the unnecessary full retraining that occurred, which we considered an error, would not profit from this measure, since no strict guidelines were designed regarding updates. The user or the experimenter decide whether they are required.

Another limiting factor in this user study is the involvement of only one participant. To minimise the impact of this factor, we have used SCED in the design of the SATMC and the user study. SCED provides guidelines for performing structured experiments involving only a small number of participants. Methods such as direct replication and the introduction of a baseline, help in lowering the impact of a low number of participants. We have implemented these two methods by incrementally changing the set of actions and by choosing a specific task that remains unchanged for the entire user study.

Finally, in its current form the tasks incorporated in the SATMC are not validated. The current paper describes a first proposal to train and assess myoelectric control. Further development of the SATMC protocol might require validation studies. For example, a Rasch analysis could verify that the task variants are indeed increasing in difficulty. Additionally, a validation study should include investigations how the outcome measures TCT and VAS are affected by the variance within a phase due to potentially different task variants between sessions.

## Conclusion

By the end of the user study P was able to achieve proportional myocontrol of four actions with a multi-articulated prosthetic hand using the incremental RR-RFF-based myocontroller. He was naive to such a control modality at the beginning of the study. Supported by the directions realised in the simultaneous training and assessment of the SATMC he succeeded in reaching a dexterous myocontrol in ADL-like tasks. The incrementality in both the myocontroller and the SATMC allowed P to progress at a level comfortable for him.

As the SATMC can be applied independent of the myocontroller, the protocol can be used in future studies to train a user in ML-based myocontrol and assess novel myocontrol approaches. This in turn will provide more validity to the SATMC and lead to results allowing for comparisons between ML-based myocontrollers.

## Data Availability

The datasets used and/or analysed during the current study are available from the corresponding author on reasonable request.

## Abbreviations

ACMC: Assessment of Capacity for Myoelectric Control
ADLs: Activities of daily living
CRT: Clothespin Relocation Test
DOFs: Degrees of freedom
EMG: Electromyography
ML: Machine-learning
P: Participant
RI: Repeatability Index
RR-RFF: Ridge Regression with Random Fourier Features
SATMC: Simultaneous Assessment and Training of Myoelectric Control
SCED: Single-Case Experimental Design
sEMG: Surface electromyography
SHAP: Southampton Hand Assessment Procedure
SI: Separability Index
TCT: Task completion time
VAS: Visual analogue scale

## Footnotes

1 For session 8 VAS evaluations by P are available, but no TCT values, see Figs. 6a and 7a. Unfortunately, the data was lost.

## References

[1] Beckerle P, Castellini C, Lenggenhager B (2019). Robotic interfaces for cognitive psychology and embodiment research: a research roadmap. WIREs Cogn Sci.

[2] Hargrove LJ, Li G, Englehart KB, Hudgins BS (2009). Principal components analysis preprocessing for improved classification accuracies in pattern-recognition-based myoelectric control. IEEE Trans Biomed Eng.

[3] Ison M, Vujaklija I, Whitsell B, Farina D, Artemiadis P (2016). High-density electromyography and motor skill learning for robust long-term control of a 7-DoF Robot Arm. IEEE Trans Neural Syst Rehabil Eng.

[4] Kapelner T, Negro F, Aszmann OC, Farina D (2018). Decoding motor unit activity from forearm muscles: perspectives for myoelectric control. IEEE Trans Neural Syst Rehabil Eng.

[5] De Luca CJ, Chang S-S, Roy SH, Kline JC, Nawab SH (2014). Decomposition of surface EMG signals from cyclic dynamic contractions. J Neurophysiol.

[6] Côté-Allard U, Campbell E, Phinyomark A, Laviolette F, Gosselin B, Scheme E (2020). Interpreting deep learning features for myoelectric control: a comparison with handcrafted features. Front Bioeng Biotechnol.

[7] Connan M, RuizRamírez E, Vodermayer B, Castellini C, Assessment of a Wearable Force- and Electromyography Device and Comparison of the Related Signals for Myocontrol. Front Neurorobot. 10 (2016). 10.3389/fnbot.2016.00017 10.3389/fnbot.2016.00017 PMC5112250 27909406

[8] Nowak M, Eiband T, Ruiz Ramírez E, Castellini C (2020). Action interference in simultaneous and proportional myocontrol: comparing force- and electromyography. J Neural Eng.

[9] Connan M, Kõiva R, Castellini C (2020). Online natural myocontrol of combined hand and wrist actions using tactile myography and the biomechanics of grasping. Front Neurorobot.

[10] Jiang X, Merhi L-K, Xiao ZG, Menon C (2017). Exploration of force myography and surface electromyography in hand gesture classification. Med Eng Phys.

[11] Castellini C, Hertkorn K, Sagardia M, González DS, Nowak M, A virtual piano-playing environment for rehabilitation based upon ultrasound imaging. In: 5th IEEE RAS/EMBS International Conference on Biomedical Robotics and Biomechatronics, pp. 548–554; 2014. 10.1109/BIOROB.2014.6913835

[12] McIntosh J, Marzo A, Fraser M, Phillips C, EchoFlex: Hand Gesture Recognition using Ultrasound Imaging. In: Proceedings of the 2017 CHI Conference on Human Factors in Computing Systems, pp. 1923–1934. ACM, Denver Colorado USA; 2017. 10.1145/3025453.3025807

[13] Zhang Y, Harrison C, Tomo: Wearable, Low-Cost Electrical Impedance Tomography for Hand Gesture Recognition. In: Proceedings of the 28th Annual ACM Symposium on User Interface Software & Technology, pp. 167–173. ACM, Charlotte NC USA; 2015. 10.1145/2807442.2807480

[14] Zhang Y, Xiao R, Harrison C, Advancing Hand Gesture Recognition with High Resolution Electrical Impedance Tomography. In: Proceedings of the 29th Annual Symposium on User Interface Software and Technology, pp. 843–850. ACM, Tokyo Japan; 2016. 10.1145/2984511.2984574

[15] Scott RN, Parker PA (1988). Myoelectric prostheses: state of the art. J Med Eng Technol.

[16] COAPT LLC: complete control system GEN2. https://coaptengineering.com/wp-content/uploads/2021/09/Coapt-Gen2-Handbook-v5.2.pdf; 2021.

[17] Ottobock: Technology for People 4.0: Ottobock at OTWorld 2018. https://www.ottobock.com/en/newsroom/news/technology-for-people-4-0-ottobock-at-otworld-2018.html. Accessed 08 Oct 2020; 2018.

[18] Jiang N, Dosen S, Muller K-R, Farina D (2012). Myoelectric control of artificial limbs—is there a need to change focus? [In the Spotlight]. IEEE Signal Process Mag.

[19] Muceli S, Jiang N, Farina D (2014). Extracting signals robust to electrode number and shift for online simultaneous and proportional myoelectric control by factorization algorithms. IEEE Trans Neural Syst Rehabil Eng.

[20] Teh Y, Hargrove LJ, Offline Repeatability Correlates with Real-Time Performance of Pattern Recognition Controllers. In: ICNR 2020, p. 2 (2020)

[21] Franzke AW, Kristoffersen MB, Jayaram V, Sluis CKVD, Murgia A, Bongers RM, Exploring the relationship between EMG feature space characteristics and control performance in machine learning myoelectric control. IEEE Transactions on Neural Systems and Rehabilitation Engineering, 1–1 (2020). 10.1109/TNSRE.2020.3029873 10.1109/TNSRE.2020.3029873 33035157

[22] Kristoffersen MB, Franzke AW, Van Der Sluis CK, Murgia A, Bongers RM (2019). The Effect of Feedback During Training Sessions on Learning Pattern-Recognition-Based Prosthesis Control. IEEE Trans Neural Syst Rehabil Eng.

[23] Nowak M, Castellini C (2016). The LET Procedure for Prosthetic Myocontrol: Towards Multi-DOF Control Using Single-DOF Activations. PLoS ONE.

[24] Nowak M, Vujaklija I, Sturma A, Castellini C, Farina D, Simultaneous and Proportional Real-Time Myocontrol of up to Three Degrees of Freedom of the Wrist and Hand. IEEE Transactions on Biomedical Engineering, 1–12 (2022). 10.1109/TBME.2022.3194104 10.1109/TBME.2022.3194104 35881594

[25] Gigli A, Gijsberts A, Castellini C (2020). The Merits of Dynamic Data Acquisition for Realistic Myocontrol. Frontiers in Bioengineering and Biotechnology.

[26] Kristoffersen MB, Franzke AW, Bongers RM, Wand M, Murgia A, Van Der Sluis CK (2021). User training for machine learning controlled upper limb prostheses: A serious game approach. J Neuroeng Rehabil.

[27] Franzke AW, Kristoffersen MB, Bongers RM, Murgia A, Pobatschnig B, Unglaube F, Van Der Sluis CK (2019). Users’ and therapists’ perceptions of myoelectric multi-function upper limb prostheses with conventional and pattern recognition control. PLoS ONE.

[28] Resnik L, Huang HH, Winslow A, Crouch DL, Zhang F, Wolk N (2018). Evaluation of EMG pattern recognition for upper limb prosthesis control: A case study in comparison with direct myoelectric control. J Neuroeng Rehabil.

[29] Kuiken TA, Miller LA, Turner K, Hargrove LJ (2016). A Comparison of Pattern Recognition Control and Direct Control of a Multiple Degree-of-Freedom Transradial Prosthesis. IEEE Journal of Translational Engineering in Health and Medicine.

[30] Scheme E, Fougner A, Stavdahl Ø, Chan ADC, Englehart K, Examining the adverse effects of limb position on pattern recognition based myoelectric control. In: 2010 Annual International Conference of the IEEE Engineering in Medicine and Biology, pp. 6337–6340. IEEE, Buenos Aires (2010). 10.1109/IEMBS.2010.5627638 10.1109/IEMBS.2010.5627638 21097173

[31] Campbell E, Phinyomark A, Scheme E (2020). Current trends and confounding factors in myoelectric control: limb position and contraction intensity. Sensors.

[32] Gijsberts A, Bohra R, SierraGonzález D, Werner A, Nowak M, Caputo B, Roa MA, Castellini CPD, Stable myoelectric control of a hand prosthesis using non-linear incremental learning. Front Neurorobot. 2014; 8. 10.3389/fnbot.2014.00008 10.3389/fnbot.2014.00008 PMC3935121 24616697

[33] Strazzulla I, Nowak M, Controzzi M, Cipriani C, Castellini C (2017). Online bimanual manipulation using surface electromyography and incremental learning. IEEE Trans Neural Syst Rehabil Eng.

[34] Schiel F, Hagengruber A, Vogel J, Triebel R Incremental learning of EMG-based control commands using Gaussian Processes. In: 4th Conference on Robot Learni, p. 10; 2020.

[35] Kyberd PJ, Aszmann OC, Farina D (2021). Outcome measures. Bionic limb reconstruction.

[36] Nowak M, Bongers RM, van der Sluis CK, Castellini C, Introducing a novel training and assessment protocol for pattern matching in myocontrol: case-study of a trans-radial amputee. In: Proceedings of MEC-Myoelectric Control Symposium. 2017.

[37] Tate RL, Perdices M, Rosenkoetter U, Shadish W, Vohra S, Barlow DH, Horner R, Kazdin A, Kratochwill T, Mcdonald S, Sampson M, Shamseer L, Togher L, Albin R, Backman C, Douglas J, Evans JJ, Gast D, Manolov R, Mitchell G, Nickels L, Nikles J, Ownsworth T, Rose M, Schmid CH, Wilson B (2016). The Single-Case Reporting Guideline In BEhavioural Interventions (SCRIBE) 2016 Statement. Phys Ther.

[38] Krasny-Pacini A, Evans J (2018). Single-case experimental designs to assess intervention effectiveness in rehabilitation: a practical guide. Ann Phys Rehabil Med.

[39] Smith JD (2012). Single-case experimental designs: a systematic review of published research and current standards. Psychol Methods.

[40] Roche AD, Vujaklija I, Amsüss S, Sturma A, Göbel P, Farina D, Aszmann OC. A structured rehabilitation protocol for improved multifunctional prosthetic control: a case study. J Visual Exp JoVE. 2015;105. 10.3791/52968 10.3791/52968 PMC4692693 26575620

[41] Segil JL, Huddle SA, Weir RF (2017). Functional assessment of a myoelectric postural controller and multi-functional prosthetic hand by persons with trans-radial limb loss. IEEE Trans Neural Syst Rehabil Eng..

[42] Simon AM, Turner KL, Miller LA, Hargrove LJ, Kuiken TA, Pattern recognition and direct control home use of a multi-articulating hand prosthesis. In: 2019 IEEE 16th International Conference on Rehabilitation Robotics (ICORR), pp. 386–391; 2019. 10.1109/ICORR.2019.8779539 10.1109/ICORR.2019.8779539 31374660

[43] Waris A, Niazi IK, Jamil M, Englehart K, Jensen W, Kamavuako EN (2019). Multiday evaluation of techniques for EMG-based classification of hand motions. IEEE J Biomed Health Inform.

[44] Ottobock.: 13E200 Electrode-Instructions for Use. 2021.

[45] Rahimi A, Recht B, Random features for large-scale kernel machines. Adv Neural Informat Process Syst. 2008; 1177–1184.

[46] Rahimi A, Recht B, Uniform approximation of functions with random bases. In: 2008 46th Annual Allerton Conference on Communication, Control, And Computing, pp. 555–561; 2008. 10.1109/ALLERTON.2008.4797607

[47] Smith LH, Kuiken TA, Hargrove LJ, Linear regression using intramuscular EMG for simultaneous myoelectric control of a wrist and hand system. In: 2015 7th International IEEE/EMBS Conference on Neural Engineering (NER), pp. 619–622; 2015. 10.1109/NER.2015.7146699

[48] Smith LH, Kuiken TA, Hargrove LJ (2016). Evaluation of linear regression simultaneous myoelectric control using intramuscular EMG. IEEE Trans Biomed Eng.

[49] Hahne JM, Biebmann F, Jiang N, Rehbaum H, Farina D, Meinecke FC, Muller K-R, Parra LC (2014). Linear and nonlinear regression techniques for simultaneous and proportional myoelectric control. IEEE Trans Neural Syst Rehabil Eng.

[50] SierraGonzález D, Castellini C, A realistic implementation of ultrasound imaging as a human–machine interface for upper-limb amputees. Front Neurorobot. 2013;7. 10.3389/fnbot.2013.00017 PMC3804922 24155719

[51] Hermansson L, Fisher A, Bernspång B, Eliasson A-C. Assessment of capacity for myoelectric control: a new rasch-built measure of prosthetic hand control. J Rehabil Med. 2004;1(1): 1–1. 10.1080/16501970410024280 10.1080/16501970410024280 16040474

[52] Light CM, Chappell PH, Kyberd PJ (2002). Establishing a standardized clinical assessment tool of pathologic and prosthetic hand function: normative data, reliability, and validity. Arch Phys Med Rehabil.

[53] Kyberd P, Hussaini A, Maillet G (2018). Characterisation of the clothespin relocation test as a functional assessment tool. J Rehabil Assist Technol Eng.

[54] Bouwsema H, Kyberd PJ, Hill W, Van Der Sluis CK, Bongers RM (2012). Determining skill level in myoelectric prosthesis use with multiple outcome measures. J Rehabil Res Dev.

[55] Bunderson NE, Kuiken TA (2012). Quantification of feature space changes with experience during electromyogram pattern recognition control. IEEE Trans Neural Syst Rehabil Eng.

[56] Nilsson N, Ortiz-Catalan M, Estimates of classification complexity for myoelectric pattern recognition. In: 2016 23rd International Conference on Pattern Recognition (ICPR), pp. 2682–2687. IEEE, Cancun (2016). 10.1109/ICPR.2016.7900040

[57] Bolstad WM, Curran JM, Introduction to Bayesian Statistics. 2017.

[58] Rasmussen CE, Williams CKI. Gaussian processes for machine learning. In: Mass: adaptive computation and machine learning. Cambridge: MIT Press; 2006.

[59] Powell MA, Kaliki RR, Thakor NV (2014). User training for pattern recognition-based myoelectric prostheses: improving phantom limb movement consistency and distinguishability. IEEE Trans Neural Syst Rehabil Eng.

[60] Nilsson N, Håkansson B, Ortiz-Catalan M (2017). Classification complexity in myoelectric pattern recognition. J Neuroeng Rehabil.

[61] Atkins DJ, Sturma A, Aszmann OC, Farina D (2021). Principles of occupational and physical therapy in upper limb amputations. Bionic limb reconstruction.

